# Co–Residence between Males and Their Mothers and Grandmothers Is More Frequent in Bonobos Than Chimpanzees

**DOI:** 10.1371/journal.pone.0083870

**Published:** 2013-12-17

**Authors:** Grit Schubert, Linda Vigilant, Christophe Boesch, Reinhard Klenke, Kevin Langergraber, Roger Mundry, Martin Surbeck, Gottfried Hohmann

**Affiliations:** 1 Department of Primatology, Max Planck Institute for Evolutionary Anthropology, Leipzig, Germany; 2 Epidemiology of highly pathogenic microorganisms, Robert Koch Institute, Berlin, Germany; 3 Department of Conservation Biology, UFZ, Helmholtz Centre for Environmental Research, Leipzig, Germany; 4 Department of Anthropology, Boston University, Boston, United States of America; University of Florence, Italy

## Abstract

In long–lived social mammals such as primates, individuals can benefit from social bonds with close kin, including their mothers. In the patrilocal chimpanzee (*Pan troglodytes* spp.) and bonobo (*Pan paniscus*), sexually mature males reside and reproduce in their natal groups and can retain post-dependency bonds with their mothers, while immatures of both sexes might also have their paternal grandmothers available. However, quantitative information on the proportion of males and immatures that co-reside with both types of these close female relatives is limited for both species. Combining genetic parentage determination and group composition data from five communities of wild chimpanzees and three communities of wild bonobos, we estimated the frequency of co-residence between (1) mature males and their mothers, and (2) immature males and females and their paternal grandmothers. We found that adult males resided twice as frequently with their mothers in bonobos than in chimpanzees, and that immature bonobos were three times more likely to possess a living paternal grandmother than were immature chimpanzees. Patterns of female and male survivorship from studbook records of captive individuals of both species suggest that mature bonobo females survive longer than their chimpanzee counterparts, possibly contributing to the differences observed in mother–son and grandmother–immature co-residency levels. Taking into account reports of bonobo mothers supporting their sons' mating efforts and females sharing food with immatures other than their own offspring, our findings suggest that life history traits may facilitate maternal and grandmaternal support more in bonobos than in chimpanzees.

## Introduction

Sociality and the maintenance of strong social bonds affect individual fitness in group–living mammals by enhancing chances for reproduction or by increasing individual or offspring survival [1-3]. Such bonds can preferentially form between relatives, driven by potential inclusive fitness gains for one or both individuals [4]. 

Long lifespans and overlapping generations in primates have the potential to provide individuals of group living species with access to a variety of kin [5]. Given the high degree of relatedness between mother and offspring, it is particularly valuable for mature offspring to maintain post-dependency bonds and receive support from their mothers. In some matrilocal societies such as baboons, for instance, social bonds between mothers and adult daughters are particularly strong and stable [6]. In addition, even grandoffspring might benefit from socialization, physical support and food provided by their grandmothers, resulting in higher infant survival or mother fertility in some human societies (reviewed in 7-9) and some non-human primates like Japanese macaques [10]. However, the availability of close kin like mothers and grandmothers varies dramatically across [5] and also within primate species [11]. Dispersal is recognized as a main determinant of patterns of kin co–residence [12]. In closely related species with similar dispersal patterns, however, adult survival is one of the key parameters of a species' life history [13] and therefore might contribute to fine–scale variation in kin availability [14]. 

As in many human societies, chimpanzee (*Pan troglodytes* spp.) and bonobo (*Pan paniscus*) females tend to leave their natal social group before reproduction [15-17]. In contrast, males are philopatric and reside with a limited number of same sex kin and potentially with their mothers [18-21]. Adolescent and adult males of both species may maintain close spatial associations and strong social bonds with their mothers, who may support their sons in agonistic interactions with other males [22-26], or influence their son’s ranging pattern within the group’s territory [27]. In addition, observations of wild bonobos suggest that males benefit from maternal support in the context of male mate competition [25,28]. As males compete for reproductive opportunities with varying degrees of success in both species (e.g. 20,29), a mother’s presence might have the potential to increase a son’s (and therefore her own) fitness.

In free–ranging populations, *Pan* females first give birth at around the age of 13–14 years [22,30,31]. Male chimpanzees may start to reproduce at 10 years of age [29], while for bonobos this information is not yet available but is expected to be comparable. Given that wild chimpanzees as well as captive bonobos and chimpanzees can live into their 50s (although average life-expectancy might be substantially lower, [32-34]), the overlap between a mother and a son’s reproductive period may be substantial and a high proportion of males might co-reside with their mothers in both species. Information on the availability of mothers to mature sons is, however, limited to anecdotal reports of individual mother–son chimpanzee pairs [22,24]. Interestingly, data from several social groups of bonobos indicate that a large proportion of mature males co-reside with their mother [20,28,35]. This hints at possible differences in the availability of mothers to mature sons, and consequently of paternal grandmothers to immatures, which ultimately might have an impact on the overall role of maternal and grandmaternal support in both species.

 In the present study we combine genetic parentage analysis and demographic information from multiple wild groups of bonobos and chimpanzees to estimate the frequency of sexually mature males co-residing with their mother and the frequency of co-residency of male as well as female immatures with their paternal grandmother. We hypothesize that patterns of co-residency differ between the two species, with mature males and immatures of both sexes being more likely to have a mother or grandmother in the group, respectively, in bonobos than in chimpanzees. We then test whether the observed differences in co-residency levels may be due to differences in life–history patterns by analyzing patterns of adult female and male survivorship from studbook records of captive animals. We used captive data because, although some survivorship data are available from multiple wild chimpanzee populations [32,36,37], comparable data are not available from the less studied bonobo. Our findings suggest the evolution of systematic differences in life history traits and co-residence patterns in the two *Pan* species.

## Methods

### Ethics statement

Permits to conduct research at Taï National Park, Côte d’Ivoire, and Salonga National Park, Democratic Republic of Congo were granted by the Ministère de la Recherches Scientifiques and the Ministère de l’Environnement et des Eaux et Forêts in Abidjan, Côte d’Ivoire, and the Institut Congolais pour la Conservation de la Nature in Kinshasa, Democratic Republic of Congo. Fecal samples from bonobos (*P. paniscus*) and West African chimpanzees (*P. t. verus*) used in this study were collected non–invasively.

### Study sites and subjects

We analyzed group composition of free–living social groups of bonobos (N=3) and chimpanzees (N=5; Table 1), in which individuals are known through long–term field investigations. Bonobo demographic data stem from the Wamba E1 social group in Luo Scientific Reserve ([16], data source: [38]), the Eyengo social group in the Lomako Forest ([39], data source: [20]) and the Bompusa social group at LuiKotale in Salonga National Park ([40], data source: present study), all within the Democratic Republic of Congo. Information on West African chimpanzees comes from the Middle and South group in Taï National Park, Côte d’Ivoire ([22], data source: [22], present study), data on East African chimpanzees (*P* *t schweinfurthii*) from Ngogo [19] and Kanyawara [41] in Kibale National Park, Uganda (data source: K. Langergraber, unpublished data; R. Wrangham, M. Muller, personal communication), and from the Kasakela social group at Gombe National Park, Tanzania ([42], data source: A. Pusey and I. Gilby, personal communication).

**Table 1:** Number of adult males, adolescent males and immatures of both sexes analyzed in bonobo and chimpanzee social groups and years examined.

**Species**	**Social group**	**Number of individuals used in the analysis**			**Years analyzed**
		Adult male*	Adolescent male*	Immature**	
Bonobo	LuiKotale	5	4	5 (12)	2008
	Lomako	6	2	7 (13)	1991–1996
	Wamba	7	1	–	1990–1991
Chimpanzee	Gombe	11	3	–	2001–2004
	Kanyawara	9	3	9 (17)	2006–2008
	Ngogo	42	28	72 (94)	2004–2010
	Middle	3	–	4 (4)	1999–2001
	South	6	9	29 (39)	2000, 2003–2007

*All individuals present were analyzed. **Only immatures for which the sire's identity was known could be analyzed. The total number of immatures present during the study periods is given in brackets.

 We included only years for which the identity and age class of all permanent residents of the social groups were known and we could also determine or had access to information on the presence of all adolescent or adult males’ mothers (see below). For the Taï chimpanzees only years for which all individuals could be genotyped and mother–son relationships confirmed or ascertained were used. We did not consider the Taï North group because by the time all members of the group were genetically characterized it had already severely declined in size as a result of disease and poaching [43]. Individuals were characterized by age classes because absolute ages were not available for individuals from all study sites, and ages of adult individuals were often estimated based on morphological criteria and include some error. For defining the age classes we applied the previously used criteria [22] for all bonobos and chimpanzees with known age estimates. Individuals were classified as follows: infant (0 - <5 years, both sexes), juvenile (5 - <10 years, both sexes), adolescent (10 - <13 year for females, 10 - <15 years for males); adult (13 years and up for females, 15 years and up for males).

### Genotyping and parentage assignment

We conducted parentage analyses for infant and juvenile individuals, and maternity assignment for adolescent and adult males for the LuiKotale bonobos and Taï chimpanzees using microsatellite genotype data. For this purpose, we non–invasively collected fecal samples from identified individuals of the LuiKotale bonobo group using the two–step ethanol–silica method [44]. Fecal sample collection from known individuals of four chimpanzee communities began in 1999 at the Taï study site, and those samples were either dried directly on silica gel, or collected using the two–step ethanol–silica method. All samples were extracted with the QIAamp DNA Stool kit (QIAGEN) with slight modifications [44], and DNA concentrations were estimated using a quantitative PCR assay [45]. 

We genotyped DNA extracts at 19 autosomal loci using a two–step amplification method described previously [46]. In brief, we combined all autosomal primer pairs [46] with template DNA in an initial multiplex PCR reaction, then used dilutions of the resultant PCR products for amplification of each individual locus using fluorescently labeled forward primers and nested reverse primers in singleplex PCR reactions. At least three replicates were required to confirm homozygous genotypes with high confidence (> 99%, [46]). We accepted heterozygous autosomal genotypes after we observed each allele in at least two independent PCR reactions. To guard against sample mix–up in the field or laboratory, we either compared individuals’ genotypes with the genotypes of individuals who were suspected from behavioral observations to be their mothers or offspring and confirmed that they shared an allele at each locus, or in cases where no suspected first–order maternal relative was available, genotyped the individual from two independently collected fecal samples. In total, we generated autosomal genotypes for 36 bonobos (97.4% complete) and 86 chimpanzees (87.6% complete). All South and Middle group Taï chimpanzees present during the years under investigation were thus successfully genotyped previously [29,47] or in the present study. Of the 33 individuals present in the LuiKotale bonobo group, 3 individuals (1 nulliparous adolescent female and 2 juveniles) could not be genotyped because no fecal samples were obtainable.

Parentage was assessed separately for chimpanzees and bonobos. We used both the exclusion method in which candidate parents are excluded by not sharing an allele at every locus with the offspring, and the likelihood approach implemented in CERVUS 3.0 [48]. Candidate fathers and mothers represented all males that were at least juvenile (5 years), and all females that were at least adolescent (10 years) at the last year of the study periods (2007 in Taï chimpanzees, 2008 in LuiKotale bonobos). We allowed for one mismatch between mother and father and the offspring to include the possibility that a mutation between parent and offspring had occurred (Table S1). We simulated genotypes for all candidate mothers (17 and 32 in bonobos and chimpanzees, respectively), all candidate fathers (13 and 36 in bonobos and chimpanzees, respectively) and 100,000 offspring using the allele frequencies from our data set. We assumed a 50% and 40% chance that candidate mothers and fathers were sampled, respectively, in the assignment simulation. Under these settings, the ratio of observed to expected assignments was similar. The proportion of genotyped loci was set to 90% (approximately matching the empirical data, see results section) and we assumed a 1% genotyping error rate. We used 99% as the strict and 95% as the relaxed confidence limits. Parentage analysis using all candidate fathers and mothers and all individuals as potential offspring were then carried out. We used a CERVUS parent–pair analysis to look for the presence of parent pairs for all individuals who were infants or juveniles at some point during the study period, and for only mothers for all males who were adolescent or adult during the entire study period.

 Parentage data for the remaining study groups were taken from ([20], Lomako Eyengo), ([38], Wamba E1) [18,27], as well as unpublished work (Langergraber, Kanyawara; 67% of all offspring genotyped). The published data set for the Wamba E1 group only detailed matrilineal relationships among adolescent and adult males and females, based on observations of maternal behavior when the respective male was infant or juvenile combined with mitochondrial DNA sequence information. Similarly, the mother was determined for all males through behavioral observation of carrying and nursing the male during infancy or juvenility for the Gombe group (A. Pusey and I. Gilby, personal communication), and confirmed through genetic parentage analysis for some individuals [49]. Those two groups were not included in all analyses requiring genetic paternity determination.

### Quantifying the presence of mothers and grandmothers

All statistical analyses were implemented in R version 2.15.0 [50]. Because adolescent males (10–15 years) have been observed to participate in male dominance interactions and because they may sire offspring [28,29,49,51], we estimated the probability of adult (*P*_adult_) as well as adolescent male (*P*_adol_) co-residency with their mothers. Co-residency was defined as the simultaneous presence of a pair of individuals (mother–son; grandmother–grandoffspring) in a social group at a given point in time. For years for which the presence or absence of the mother was known for all males (Table 1), we calculated *P*_adult_ and *P*_adol_ as: *Years co-residing with mother in respective age class* (*adult, adolescent*) */ Total years in respective age class* (*adult, adolescent*). For each species, we then averaged *P*_adult_ and *P*_adol_ over all adult or adolescent males, respectively. For each social group we averaged *P*_adult_ and *P*_adol_ over all adult or adolescent males from the respective social group. We report the number of adult and adolescent males used in total for each species and social group in Table 1.

The probabilities for immatures (infants and juveniles) to co-reside with their paternal grandmother (*P*_immat_) and the numbers of immatures used in the analyses per species and per social group (Table 1) were calculated in a similar way. The data set was restricted to immatures for which a father was determined. This was necessary because if the identity of the father was not known (in cases of extra-group paternities or when not all potential fathers present at the time of conception were sampled), we had no means to infer paternal grandmaternal relationships. We did not investigate the presence of maternal grandmothers because we were only interested in potential effects of mothers on the reproductive success of their sons and the benefits paternal grandmothers might provide towards their son’s offspring. Also, most females of both species disperse from their natal group upon adolescence [15,16]; thus, individuals rarely reside in the same social group as their maternal grandmother.

We used Kruskal–Wallis H–tests and Mann–Whitney U–tests to examine differences in the proportion of adult and adolescent males having a mother or immatures having a paternal grandmother, between i) social groups within species and ii) between species. Mann–Whitney U–tests were exact throughout [6] and conducted using the function wilcox.exact of the R–package exactRankTests [52]. 

The Taï communities [9] as well as Gombe [53] have undergone episodes of disease outbreaks, which might have influenced group demography and potentially the outcome of comparisons between species. To examine this possibility, we re-ran all analyses excluding Taï Middle, Taï South and Gombe.

### Correlation between group size and the presence of mothers or grandmothers

The total number of individuals may affect quantitative patterns of relatedness within social groups, where, on average, higher proportions of related individuals might reside in smaller groups [54]. As our study communities varied considerably in size, we tested for potential correlations between communities’ mean *P*_adult_, *P*_adol_ and *P*_immat_ and mean numbers of adult and adolescent individuals per social group, respectively, using exact Spearman rank correlations. These were conducted using an R–function written by R. Mundry.

### Adult survivorship

Species differences in the survival probabilities of mature individuals are a potential source of variation in co-residency frequencies within groups. We thus explored female as well as male survivorship among adolescent and adult bonobos and chimpanzees. As appropriate data stemming from wild bonobos are currently not available, we turned to studbook records of captive bonobos ([33], current as of 2007) and captive chimpanzees ([34], current as of 2006). Only individuals with known dates of birth, death (if applicable), and status (alive or dead) at the end of the studbook study period (01.01.2007 for bonobos, 31.12.2006 for chimpanzees) where considered. We used exact birth and death dates whenever possible. If birth or death dates of individuals were known only to the month, we set the date to the 15^th^ of that month. When comparing both species we further excluded chimpanzees that were born more than five years before the first bonobo female or male, respectively, to ensure that conditions of keeping and veterinary services were as compatible as possible. As we were interested in the survival of potentially reproducing individuals, we included only individuals that had reached the age of 9, the minimum age at which females of both species have been reported to give birth [55]. Nine was thus the starting age in the analysis, with all individuals starting with equal probabilities to survive.

 To determine which factors predict survival, we used a Cox mixed–effects model (package 'coxme' in R, [56,57]) on females and males separately for each species (Model I) and separately on either females or males of both species (Model II). The response variable was the hazard function, the death rate at time *t* conditional on survival until time *t*. We included the following predictor variables as fixed effects:

• *Sex*. To validate whether sex–differences in survival among captive individuals reflect the pattern expected or observed in the wild [32], we included this variable into Model I.
• *Species*. As our objective was to examine differences in survival between bonobos and chimpanzee, we included this variable into Model II.
• *Location status*. Although from the records no deaths were attributable to biomedical research, we controlled for the potential detrimental effects of being temporarily housed in a research facility by including a binary variable defining two types of rearing conditions: (1) always housed in zoological, non-research institutions, or (2) having spent at least some time in biomedical or private facilities.
• *Birth*. Pregnancy, parturition and lactation may affect female survival [50]. Therefore, in the analysis of female survival in Model II, we incorporated the number of births given as time–dependent variable by creating separate time intervals for subsequent birth events.
• *Transfer*. Transfers between different locations (i.e., when being captured or when being moved between zoological institutions) are most likely stressful events with potential impact on the survival of an individual. We incorporated *Transfer* as time–dependent variable in the same manner as birth.
• *Entry age*. We also controlled for the age of individuals at which the time–dependent events of giving *birth* or being *transferred* occurred.

Individual identity was incorporated as a random effect. Sample size for bonobos in terms of individuals and deaths observed was small as compared to chimpanzees. We therefore did not examine *P* – values for all co-variates from the Cox model, but examined whether female and male survival (Model I) or bonobo and chimpanzee survival (Model II) differed by permuting the *Sex* or *Species* assignment to individuals 1000 times, including the original data set as one permutation [58]. 

The survivorship function describing the probability of surviving discrete time intervals was plotted for each model from the original data without controlling for co-variates (package 'survival' in R, [59]). To illustrate how survival among captive chimpanzees relates to estimates from free–living populations, we also plotted the survival curve from the synthetic life table of wild chimpanzees published by [32] alongside our estimate of captive chimpanzee survival.

## Results

### Parentage assignment

We assessed the parentage of 18 bonobos and 51 chimpanzees, and of these attributed mothers to 15 bonobos and 45 chimpanzees (Table S1). This includes maternity assignment for six bonobo and ten chimpanzee males that were adolescent or adult during the study period. Maternal relationships inferred from behavioral observations were confirmed for all genotyped individuals who were observed as infants or juveniles. Fathers were determined for five LuiKotale bonobo and 30 Taï chimpanzee offspring (Table S1). 

 All non-assigned candidate parents present during the study period were excluded by at least two mismatches to the genotypes of the individual under investigation (range 2 - 8), and individual probabilities of excluding non-parents were close to 1 (0.98419 - 1). All but one assignment met the 99% confidence criterion, and in this single case the 95% confidence criterion was met (Table S1). In total, five individual mismatches between an offspring and an assigned parent (three times between sire and offspring, two times between mother and offspring; see Table S1 for more detailed information) were observed, most likely due to germ line mutations [60]. 

### Quantifying the presence of mothers and grandmothers

**Figure 1:**
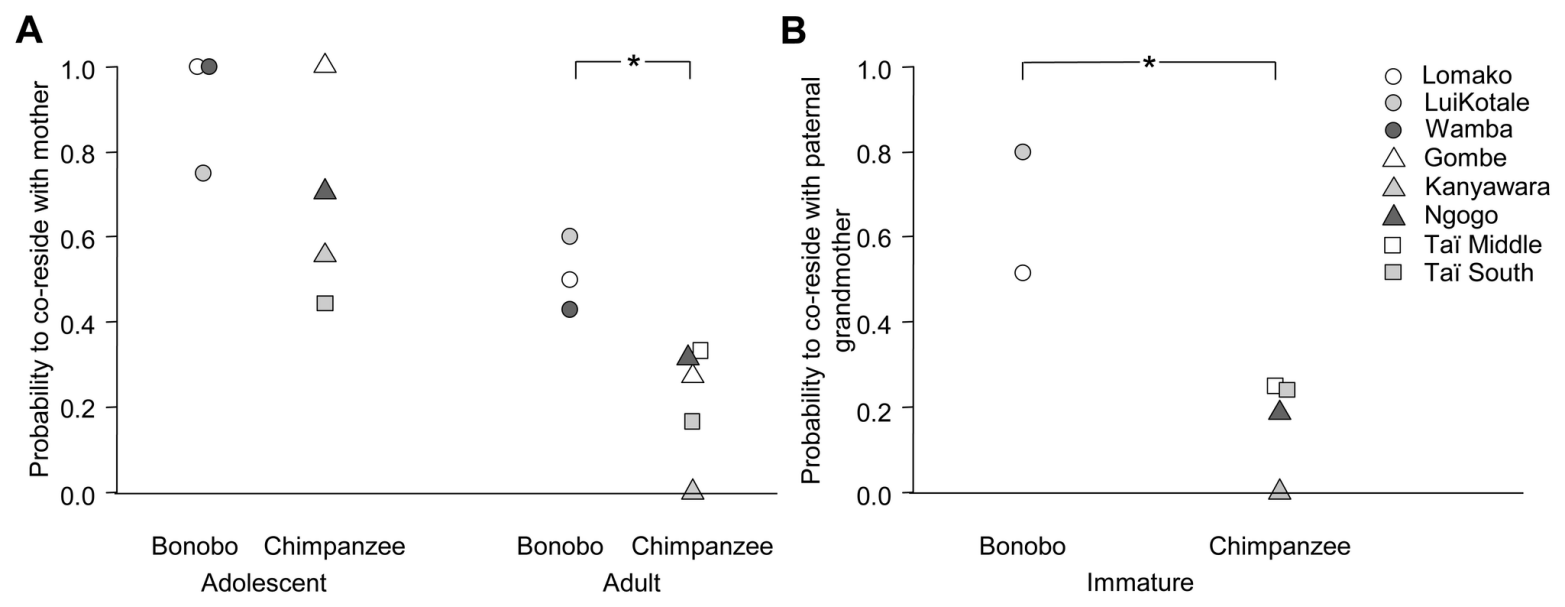
Probabilities of co-residence with mothers and grandmothers in bonobo and chimpanzee groups. Probabilities of (A) adolescent and adult male co-residency with their mother and (B) offspring co-residency with their paternal grandmother in social groups of free–living bonobos and chimpanzees. (A) When comparing both species, adult bonobo males had higher probabilities to live together with their mothers as compared to chimpanzees. No adolescent male was present in Taï Middle during our study period. (B) Estimated from all offspring with determined paternity, bonobo offspring had a higher chance to co-reside with their paternal grandmothers than observed in chimpanzees. *P – value from Mann–Whitney U–test < 0.05.

Adult male bonobos had a higher probability to co-reside with their mothers (0.50, N_males_ = 18) than did adult male chimpanzees (0.26, N_males_ = 71; Mann–Whitney U–test: U = 458, *P* = 0.030; see Figure 1A for mean *P*_adult_ per social group). Although adolescent bonobo males had a higher probability to co-reside with their mothers compared to adolescent male chimpanzees (bonobo: 0.86, N_males_ = 7; chimpanzee: 0.66, N_males_ = 43; see Figure 1A for mean *P*_adol_ per social group), this difference was not statistically significant (Mann–Whitney U–test: U = 118, *P* = 0.276). Within each species, the mean fraction of males co-residing with mothers did not differ among social groups (adult bonobos: Kruskal–Wallis H–test: χ^2^ = 0.618, df = 2, *P* = 0.734; adult chimpanzees: χ^2^ = 5.224, df = 4, *P* = 0.265; adolescent bonobos: χ^2^ = 0.750, df = 2, *P* = 0.687; adolescent chimpanzees: χ^2^ = 4.570, df = 3, *P* = 0.206; see Table 1 for mean number of males per social group). 

Immature males and females were more likely to possess a living paternal grandmother in bonobos (0.63, N_offspring_ = 12) than in chimpanzees (0.19, N_offspring_ = 114; Mann–Whitney U–test: U = 337, *P* < 0.001; see Figure 1B for mean *P*_immat_ per social group). Again, there were no differences among social groups within species (bonobos: Mann–Whitney U–test: U = 8, *P* = 0.104; chimpanzees: χ ^2^ = 2.707, df = 3, *P* = 0.439; see Table 1 for mean number of immatures per social group).

We found very similar results even when we excluded the three chimpanzee groups (Taï Middle, Taï South and Gombe) that had experienced anthropogenically induced population declines. The probability of mature males to have a mother in the group was higher in bonobos than chimpanzees, with this difference achieving statistical significance for adults (bonobos: *P*_adult_ = 0.50, N_males_ = 18; chimpanzees: *P*_adult_ = 0.26, N_males_ = 51; Mann–Whitney U–test: U = 330.5, *P* = 0.044) but not for adolescents (bonobos: *P*_adol_ = 0.86, N_males_ = 7; chimpanzees: *P*_adol_ = 0.69, N_males_ = 31; Mann–Whitney U–test: U = 87.5, *P* = 0.334). Also, the probability of immatures to co-reside with their paternal grandmother was significantly higher in bonobos as compared to chimpanzees (bonobos: *P*_immat_ = 0.63, N_males_ = 12; chimpanzees: *P*_immat_ = 0.17, N_males_ = 81; Mann–Whitney U–test: U = 223.5, *P* = < 0.001).

Neither the probability of males co-residing with mothers nor the probability of immatures co-residing with grandmothers was correlated with the average numbers of adult and adolescent individuals present in the social group (Spearman’s rank correlations; adult males: ρ = –0.452, N_groups_ = 8, *P* = 0.267; adolescent males: ρ = –0.148, N_groups_ = 7, *P* = 0.781; immatures: ρ = –0.657, N_groups_ = 6, *P* = 0.175).

### Adult survival in captive populations

**Figure 2:**
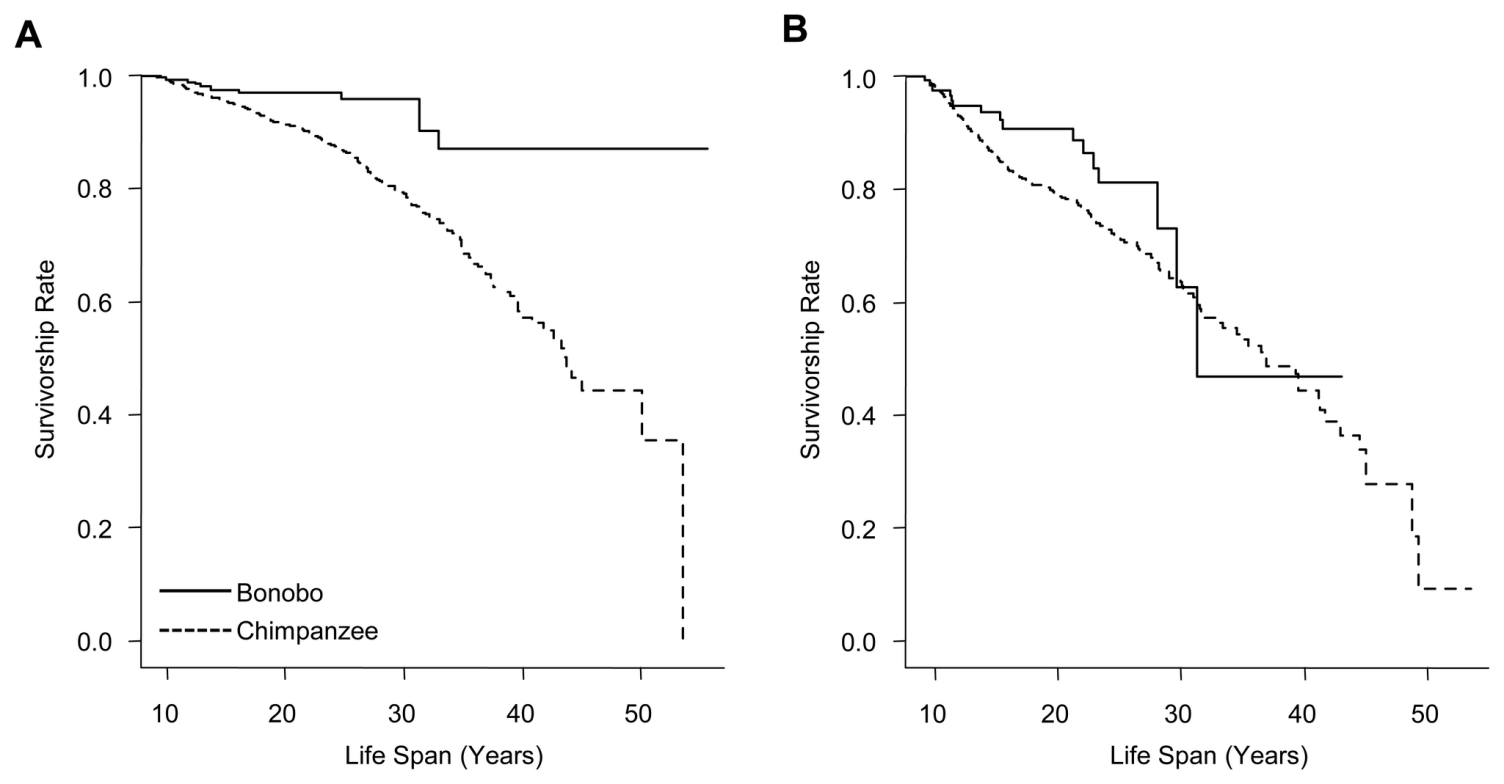
Female and male survivorship in captive bonobos and chimpanzees. The survivorship function is the probability of surviving discrete time intervals. (A) Adult captive bonobo females had significantly higher rates of survival than captive chimpanzee females (*P* – value from permutation procedure = 0.011). (B) Among adult males, bonobos showed only a trend to survive better then chimpanzees (*P* – value from permutation procedure = 0.060).

Captive adult female bonobos experienced higher survival than did captive adult female chimpanzees (*P* = 0.011; Figure 2A, Table 2). Similarly, captive male bonobos tended to survive better than captive male chimpanzees, but here the difference showed only a trend towards statistical significance (*P* = 0.060; Figure 2B, Table 2).

**Table 2:** Results from a Cox mixed–effects model examining survival in captive female and male bonobos and chimpanzees.

	Estimate*	Std. Error	*z*	*P* – value
**Female survival**				
Location status	–0.03	0.32	–0.09	
Species**	1.17	0.63	1.84	0.011
Birth	0.05	0.13	0.38	
Transfer	–0.08	0.07	–1.11	
Entry age	–0.07	0.02	–3.30	
**Male survival**				
Location status	–0.01	0.17	–0.06	
Species***	0.44	0.30	1.48	0.060
Transfer	0.03	0.07	0.44	
Entry age	–0.06	0.02	–3.43	

*Species* (chimpanzee female; bonobo female), *Location status* (permanently housed in zoological facilities; non-permanently housed in zoological facilities), *Birth* (number and timing of individuals' giving birth; only in the female model), *Transfer* (number and timing of individuals' transfers between locations) and *Entry age* (age of individuals when entering each time interval between subsequent transfers or, for females, transfers and births) were included as fixed (the three latter ones time–dependent) effects. A P – value for *Species* was derived from a permutation procedure.

*Estimate of the regression coefficient. **Samples sizes: bonobo = 329, bonobo deaths = 11; chimpanzee = 2378, chimpanzee deaths = 263. ***Samples sizes: bonobo =129, bonobo deaths = 16; chimpanzee = 768, chimpanzee deaths = 191.

In chimpanzees, captive adult females experienced higher survival than males (*P* = 0.001; Figure S1B, Table S2). Females also tended to have higher survival than males in bonobos, but the difference was not statistically significant (*P* = 0.081; Figure S1A, Table S2). Although the different forms of data make direct comparisons untenable, it appears that as expected captive chimpanzees have higher survival than those in the wild, and under both settings females experience higher survival than males (illustrated in Figure S1B).

## Discussion

In a comparison of social group composition in wild chimpanzees and bonobos, two closely related male philopatric primates, we found an increased probability of co-residence of adult males with their mothers and of co-residence of immatures with their paternal grandmothers, in bonobos compared to chimpanzees. Analyzing studbook records from captivity, we found that higher survival, particularly among bonobo females, could explain these findings. 

Adult male bonobos were significantly more likely to co-reside with their mothers than were adult male chimpanzees (Figure 1A). Also, immature bonobo females and males lived significantly more often in the presence of their paternal grandmother than did immature chimpanzees (Figure 1B). We did not find significant differences in patterns of co–residence among social groups within each species, although variation was large (Figure 1). Patterns of co-residence were independent of the size of the social group. Our examination of records on captive animals showed that female bonobos of reproductive age had a higher survival rate than chimpanzee females (Figure 2A, Table 2), and suggested a trend to higher survival among adolescent and adult bonobo males compared to chimpanzees males (Figure 2B, Table 2). The small number of individuals that had already reached an old age in bonobos (i.e., above 30 years) prevented quantitative analysis of life expectancy. Yet, our results suggest that in chimpanzees fewer individuals, particularly females, reach older ages and are thus less often present for an extended period of a son’s reproductive life.

Co-residence with the mother has been shown to be advantageous in several matrilocal cercopithecine primates, where mothers might be present during extended periods of their daughters’ reproductive lives [10] and females can gain higher reproductive success if their mothers are present [10,61-63]. Similarly, sons who associate strongly with their mothers in the patrilocal egalitarian northern Muriqui new world monkeys are among the most reproductively successful males [64]. Social dominance has a large effect on chimpanzee male reproductive success, and short–term reproduction is skewed [29,49,51]. The most successful sires also have a high social status in the Lomako bonobos [20], and male dominance ranks translate into mating success in the LuiKotale bonobo group [28]. In the Wamba bonobo group, the presence of dominant mothers may help to increase the dominance rank of their sons [25,26]. Behavioral evidence from LuiKotale further indicates that mothers exert a positive effect on their sons’ mating success and reduce the influence of male dominance upon mating. Together, these results suggest that female bonobos can effectively compete for indirect fitness benefits by increasing their sons’ reproduction [25,28]. Considering that the oldest male in our data set that co-resided with his mother was a bonobo, estimated to be 27–34 years of age during the study period [65], a high incidence of maternal presence at least in this species could render maternal support an important factor in male mate competition. How mating translates into reproductive success of males in bonobos as compared to chimpanzees remains to be tested in future studies. This highlights the importance of the collection of long–term group composition and relatedness data on *Pan* for elucidating the impact of mothers on their offspring’s reproductive strategies. 

A higher incidence of mother–son co-residence in bonobos as compared to chimpanzees might also be related to differences in cognitive development. While maternal presence in bonobos has been shown to positively affect a juvenile son’s development of socio-emotional competence [66], studies by Wobber and colleagues indicate that certain facets of bonobo psychology are developmentally delayed compared to chimpanzees [67,68], and that this in turn may be related to higher tolerance in bonobos than chimpanzees [69]. Furthermore, male bonobos react to social challenges in a less status–striving way than do male chimpanzees [70], which might be important for the persistence of strong mother–son bonds into the adulthood of the sons. While the self-domestication hypothesis links changes in the cognitive development of bonobos to selection against aggression [71], further studies will be required to address the interplay between these long–lasting bonds in bonobos and the underlying psychology of the species.

The higher rates of survival among sexually mature female bonobos than female chimpanzees found here might contribute to explaining a higher availability of mothers to adult male bonobos in the wild. Adult survivorship in nature is likely constrained by a variety of factors that may have differentiated effects on females and males, including resource abundance, population density and predation [13,72]. Thus conclusions on survivorship drawn from data from captivity should be regarded with caution and would benefit by validation using data from the wild, once those become available. Nevertheless, adapted, physiological determinants of life span can result in congruent patterns under both wild and captive living conditions [73]. In line with this argument, although chimpanzees in captivity experience overall lower rates of early adult mortality as compared to natural populations, in both wild and captive populations adult mortality is higher and senescence is more rapid as compared to traditional human societies [32,74]. The captive data used in this study are from bonobos and chimpanzees that should experience a high degree of health care (medical facilities and private pet traders were excluded from the analysis) and no food limitations. Such conditions may be expected to attenuate or mask differences in adult survivorship. Yet females in both species appeared to have higher rates of age specific survival than males (Figure S1, Table S2). This is consistent with previous reports from the wild and a captive chimpanzee study [32,36], and the expected pattern under the polygynous mating system of both species [75]. While a direct comparison of survival of individuals living in different conditions (captive versus wild) is inappropriate, a comparison under similar conditions has the potential to provide insights into biological differences between species. Accordingly, the significant difference in adult female survivorship between bonobos and chimpanzees found here seems a plausible explanation for the high frequency of adult male co-residency with their mothers in wild bonobo but not in wild chimpanzee communities.

There are several possible explanations for why female bonobos live longer than female chimpanzees. One is suggested by classical theories of inter-specific variation in longevity, which predict that species that experience low mortality rates from external factors such as predation will evolve a later onset of senescence [76,77]. Gregariousness in primates is regarded as strategy to reduce predation pressure [6,52]. Female bonobos have been proposed to be more gregarious than female chimpanzees [78], although variation among chimpanzees throughout their range is large [79]. Higher gregariousness in bonobo females might have reduced extrinsic mortality from predation, resulting in stronger selection for a longer life as compared to chimpanzee females. 

Higher survival of female bonobos compared to female chimpanzees, by translating into higher indirect fitness benefits for females due to a bigger effect on their sons’ reproductive success, could explain the species difference in co-residency of male and female immatures and grandmothers. Bonobo immatures were, on average, more than three times more likely to co-reside with their paternal grandmothers as compared to chimpanzee offspring in our study (with an average frequency of 0.63 in bonobos versus 0.19 in chimpanzees). In humans, grandmaternal provisioning and care towards grandoffspring can enhance the survival of children, thereby increasing a grandmother’s indirect fitness (e.g. Ethiopia: [80], The Gambia: [81]) and presumably shaping the evolution of female life history (grandmother hypothesis, [82]). Such grandmaternal behavior is usually directed towards the offspring of daughters in humans ([7], but see 83) and also exists in some matrilocal cercopithecine primates [10,84,85]. While there is an ongoing debate whether paternal grandmothers recognize their grandoffspring in species with a promiscuous mating system ([86], but see 87) western chimpanzee sires tend to play more frequently with their own offspring [88]. And while paternal care has not been shown in East African chimpanzees, in at least one social group males are more likely to associate with and have similar ranging patterns to females with whom they have produced offspring [27]. In addition, captive chimpanzees perceive similarities in the faces of related but unfamiliar individuals, giving evidence for visual kin recognition in this species [89]. This suggests that, as in other primates living in multi-male, multi-female groups [11,90], there are mechanisms of paternal kin recognition in the genus *Pan*, which are a prerequisite for paternal grandmaternal investment. Both bonobo and chimpanzee females do provide benefits to their own immature offspring by sharing plant food (summarized in [91]), but only bonobo females provision the young of other females by offering orally or manually processed high priced food items such as fruit and meat [91,92]. This hints that there might be some potential for grandmaternal care in bonobos, but in the absence of data on differentiated grandmaternal behavior (e.g. playing, food sharing, grooming) towards offspring and grandoffspring, the discussion of a potentially adaptive value of such behavior remains rather speculative. Interestingly, limited data from wild populations of both species indicate that overall infant mortality may be substantially lower in bonobos [55], but thus far information on whether grandmother presence improves infant survival (as observed in humans) is not available.

In sum, higher survival, particularly among females, possibly contributes to the increased frequency of co-residence of wild adult males with their mothers and of co-residence of immatures with their paternal grandmothers, in bonobos compared to chimpanzees. Our results suggest that fundamental aspects of life history such as differences in survival between closely related species can indeed become apparent under a controlled captive setting and merit further scrutiny in natural populations, once those data become available for bonobos. In the light of results from behavioral studies on agonistic support by mothers of mating attempts of their sons and food sharing with immatures by females, our results suggest that bonobos may show adaptations in life history traits that allow for a higher degree of maternal and grandmaternal support than in chimpanzees.

## Supporting Information

Figure S1 **(**A**) Bonobo and (**B**) chimpanzee survivorship among captive females and males.** In (B), for comparison, survivorship rates of wild chimpanzees published previously [32]are drawn in grey. In both species, captive females had higher rates of survival than captive males, however the difference was statistically significant only in chimpanzees (*P* – value from permutation procedure = 0.001), but not among bonobos (*P* – value from permutation procedure = 0.081).
(TIF)

Table S1 **Parentage assignments in the free–living LuiKotale Bompusa bonobo group and the Taï Middle and South Western chimpanzee groups.** Parentage was determined from genotypes comprised of 19 autosomal loci. As our chimpanzee study spans several years, the age class of an individual might have changed over time. Maternal relationships known from behavior were confirmed in all infants and juveniles. All but one assignment met the 99% confidence criterion (see footnote). Females are written in capitals. n.a., not assigned.
^1^ Reported only for male offspring being adolescent or adult in study period ^2^; Reported for parent – pair comparisons with confirmed or assigned mother ^3^; One adolescent female and two juveniles were not genotyped ^4^; One mismatch (1 base pair indel common in that population) to assigned sire ^5^; One mismatch to mother at heterozygous locus (both alleles differ, one allele with one repeat unit difference to maternal allele), in triadic comparison with potential sires one mismatch to the assigned sire at heterozygous locus (one repeat unit difference to one paternal allele) ^6^; Paternity assigned at 95% confidence level ^7^; In triadic comparison one mismatch to the assigned sire at heterozygous locus (one repeat unit difference) ^8^; One mismatch to mother at heterozygous locus (both alleles differ, one allele with one repeat unit difference to maternal allele).
(DOCX)

Table S2 **Results from a Cox mixed–effects model examining survival in captive female and male bonobos and chimpanzees.** *Sex* (female; male), *Location* *status* (permanently housed in zoological facilities; non-permanently housed in zoological facilities), *Transfer* (number and timing of individuals' transfers between locations) and *Entry* *age* (age of individuals when entering each time interval between subsequent transfers) were included as fixed effects (the two latter time–dependent). A P – value for *Sex* was derived from a permutation procedure.
*Estimate of the regression coefficient. **Samples sizes: female = 329, female deaths = 11; male = 129, male deaths=16. ***Samples sizes: female = 2427, female deaths = 287; male = 811, male deaths = 220.
(DOCX)

## References

[1] Schülke O, Bhagavatula J, Vigilant L, Ostner J (2010) Social Bonds Enhance Reproductive Success in Male Macaques. Curr Biol 20: 2207-2210. doi:10.1016/j.cub.2010.10.058. PubMed: 21093261. 21093261

[2] Silk JB, Beehner JC, Bergman TJ, Crockford C, Engh AL et al. (2010) Strong and consistent social bonds enhance the longevity of female baboons. Curr Biol 20: 1359-1361. doi:10.1016/j.cub.2010.05.067. PubMed: 20598541. 20598541

[3] Cameron EZ, Setsaas TH, Linklater WL (2009) Social bonds between unrelated females increase reproductive success in feral horses. Proc Natl Acad Sci U S A 106: 13850-13853. doi:10.1073/pnas.0900639106. PubMed: 19667179. 19667179 PMC2728983

[4] Hamilton WD (1964) The genetical evolution of social behaviour II. J Theor Biol 7: 17-52. doi:10.1016/0022-5193(64)90039-6. PubMed: 5875340. 5875340

[5] Strier KB (2008) The effects of kin on primate life histories. Annu Rev Anthropol 37: 21-36. doi:10.1146/annurev.anthro.37.081407.085218.

[6] van Schaik CP (1989) The ecology of social relationships amongst female primates. In: Standen V Foley R Comparative socioecology: The behavioural ecology of humans and other mammals. Oxford: Blackwell pp. 195-218.

[7] Sear R, Mace R (2008) Who keeps children alive? A review of the effects of kin on child survival. Evol Hum Behav 29: 1-18. doi:10.1016/j.evolhumbehav.2007.10.001.

[8] Voland E, Chasiotis A, Schiefenhoevel W (2005) Grandmotherhood – the Evolutionary Significance of the Second Half of Female Life Voland E Chasiotis A Schiefenhoevel W New Brunswick, NJ: Rutgers University Press.

[9] Strassmann BI, Garrard WM (2011) Alternatives to the grandmother hypothesis. Hum Nature 22: 201-222. doi:10.1007/s12110-011-9114-8. 22388808

[10] MacDonald Pavelka MS, Fedigan LM, Zohar S (2002) Availability and adaptive value of reproductive and postreproductive Japanese macaque mothers and grandmothers. Anim Behav 64: 407-414. doi:10.1006/anbe.2002.3085.

[11] Buchan JC, Alberts SC, Silk JB, Altmann J (2003) True paternal care in a multi-male primate society. Nature 425: 179-181. doi:10.1038/nature01866. PubMed: 12968180. 12968180

[12] Greenwood PJ (1980) Mating systems, philopatry and dispersal in birds and mammals. Anim Behav 28: 1140-1162. doi:10.1016/S0003-3472(80)80103-5.

[13] Stearns SC (1992) The Evolution of Life History Theories. New York: Oxford University Press.

[14] Altmann S, Altmann J (1979) Demographic constraints on behavior and social organization. In: Bernstein IS Smith EO Primate Ecology and Human Origins: Ecological Influences on Social Organization New York: Garland STPM Press

[15] Pusey AE (1979) Intercommunity transfer of chimpanzees in Gombe National Park. In: Hamburg D McCown E The Great Apes Menlo Park: Benjamin/Cummings. pp. 465–479

[16] Kano T (1982) The social group of pygmy chimpanzees (*Pan* *paniscus*). Primates 23: 171–188. doi:10.1007/BF02381159.

[17] Murdock GP (1981) Atlas of World Cultures. University of Pittsburgh Press.

[18] Langergraber KE, Mitani JC, Vigilant L (2007) The limited impact of kinship on cooperation in wild chimpanzees. Proc Natl Acad Sci U S A 104: 7786-7790. doi:10.1073/pnas.0611449104. PubMed: 17456600. 17456600 PMC1876525

[19] Mitani JC, Watts DP, Pepper JW, Merriwether DA (2002) Demographic and social constraints on male chimpanzee behaviour. Anim Behav 64: 727-737. doi:10.1006/anbe.2002.4014.

[20] Gerloff U, Hartung B, Fruth B, Hohmann G, Tautz D (1999) Intracommunity relationships, dispersal pattern and paternity success in a wild living community of Bonobos (*Pan* *paniscus*) determined from DNA analysis of faecal samples. Proc Biol Sci 266: 1189-1195. doi:10.1098/rspb.1999.0762. PubMed: 10406131. 10406131 PMC1689947

[21] Hashimoto C, Tashiro Y, Hibino E, Mulavwa M, Yangozene K et al. (2008) Longitudinal structure of a unit-group of bonobos: male philopatry and possible fusion of unit-groups. In: Furuichi T Thompson J The Bonobos: Behavior, Ecology and Conservation. New York: Springer pp. 107-119.

[22] Boesch C, Boesch-Achermann H (2000) The Chimpanzees of the Taï Forest: Behavioural Ecology and Evolution. Oxford: Oxford University Press.

[23] Boesch C (2009) The Real Chimpanzee: Sex Strategies in the Forest. Cambridge: Cambridge University Press.

[24] van Lawick-Goodall J (1967) Mother–offspring relationships in free-ranging chimpanzees. In: Morris D Primate Ethology. London: Weidenfeld and Nicolson pp. 287–346.

[25] Furuichi T (1997) Agonistic interactions and matrifocal dominance rank of wild bonobos (*Pan* *paniscus*) at Wamba. Int J Primatol 18: 855-875. doi:10.1023/A:1026327627943.

[26] Furuichi T (2011) Female contributions to the peaceful nature of bonobo society. Evol Anthropol 20: 131-142. doi:10.1002/evan.20308. PubMed: 22038769. 22038769

[27] Langergraber KE, Mitani JC, Watts DP, Vigilant L (2013) Male–female socio-spatial relationships and reproduction in wild chimpanzees. Behav Ecol Sociobiol: 1-13.24436508

[28] Surbeck M, Mundry R, Hohmann G (2011) Mothers matter! Maternal support, dominance status and mating success in male bonobos (*Pan* *paniscus*). Proc R Soc of London B 278: 590-598. PubMed: 20810444. 10.1098/rspb.2010.1572 PMC3025686 20810444

[29] Boesch C, Kohou G, Néné H, Vigilant L (2006) Male competition and paternity in wild chimpanzees of the Taï Forest. Am J Phys Anthropol 130: 103-115. doi:10.1002/ajpa.20341. PubMed: 16353223. 16353223

[30] Nishida T, Corp N, Hamai M, Hasegawa T, Hiraiwa-Hasegawa M et al. (2003) Demography, female life history, and reproductive profiles among the chimpanzees of Mahale. Am J Primatol 59: 1098-2345. PubMed: 12619045. 10.1002/ajp.10068 12619045

[31] Kuroda S (1989) Developmental retardation and behavioral characterisitcs of pygmy chimpanzees. In: Heltne PG Marquardt AE Understanding Chimpanzees. Chicago: Chicago Academy of Sciences pp. 184–193.

[32] Hill K, Boesch C, Goodall J, Pusey A, Williams J et al. (2001) Mortality rates among wild chimpanzees. J Hum Evol 40: 437-450. doi:10.1006/jhev.2001.0469. PubMed: 11322804. 11322804

[33] Perebom ZJJM, Stevens JMG (2008) International studbook for the bonobo *Pan**paniscus* SCHWARZ 1929. Belgium: Royal Zoological Society of Antwerp.

[34] Carlsen F (2007) European studbook for the chimpanzee *Pan**troglodytes*. Copenhagen Zoo, Roskildevej 38, PO Box 7, DK-2000 Frederiksberg, Denmark

[35] Furuichi T, Ihobe H (1994) Variation in male relationships in bonobos and chimpanzees. Behaviour 130: 211-228. doi:10.1163/156853994X00532.

[36] Dyke B, Gage TB, Alford PL, Swenson B, Williams-Blangero S (1995) Model life table for captive chimpanzees. Am J Primatol 37: 25-37. doi:10.1002/ajp.1350370104. 32005046

[37] Emery Thompson M, Jones JH, Pusey AE, Brewer-Marsden S, Goodall J et al. (2007) Aging and fertility patterns in wild chimpanzees provide insights into the evolution of menopause. Curr Biol 17: 2150-2156. doi:10.1016/j.cub.2007.11.033. PubMed: 18083515. 18083515 PMC2190291

[38] Hashimoto C, Takenaka O, Furuichi T (1996) Matrilineal kin relationship and social behavior of wild bonobos (*Pan* *paniscus*): Sequencing the D-loop region of mitochondrial DNA. Primates 37: 305-318. doi:10.1007/BF02381862.

[39] Susman RL (1984) The Pygmy Chimpanzee: Evolutionary Biology and Behavior. New York: Plenum Press.

[40] Hohmann G, Fruth B (2003) LuiKotal – a new site for field research on bonobos in the Salonga National Park. Pan African NEWS 10: 25–27.

[41] Wrangham RW, Chapman CA, Clark-Arcadi AP, Isabirye-Basuta G (1996) Socio-ecology of Kanyawara chimpanzees: implications for understanding the costs of great ape groups. In: McGrew WC Marchant LF Nishida T Great Ape Societies. Cambridge: Cambridge University Press pp. 45–57.

[42] Goodall J (1986) The Chimpanzees of Gombe. Cambridge: Harvard University Press.

[43] Boesch C, Crockford C, Herbinger I, Wittig R, Moebius Y et al. (2008) Intergroup conflicts among chimpanzees in Taï National Park: lethal violence and the female perspective. Am J Primatol 70: 519-532. doi:10.1002/ajp.20524. PubMed: 18214941. 18214941

[44] Nsubuga AM, Robbins MM, Roeder AD, Morin PA, Boesch C et al. (2004) Factors affecting the amount of genomic DNA extracted from ape faeces and the identification of an improved sample storage method. Mol Ecol 13: 2089-2094. doi:10.1111/j.1365-294X.2004.02207.x. PubMed: 15189228. 15189228

[45] Morin PA, Chambers KE, Boesch C, Vigilant L (2001) Quantitative polymerase chain reaction analysis of DNA from noninvasive samples for accurate microsatellite genotyping of wild chimpanzees (*Pan* *troglodytes* *verus*). Mol Ecol 10: 1835-1844. doi:10.1046/j.0962-1083.2001.01308.x. PubMed: 11472550. 11472550

[46] Arandjelovic M, Guschanski K, Schubert G, Harris TR, Thalmann O et al. (2009) Two-step multiplex polymerase chain reaction improves the speed and accuracy of genotyping using DNA from noninvasive and museum samples. Mol Ecol Resour 9: 28-36. doi:10.1111/j.1755-0998.2008.02387.x. PubMed: 21564562. 21564562

[47] Vigilant L, Hofreiter M, Siedel H, Boesch C (2001) Paternity and relatedness in wild chimpanzee communities. Proc Natl Acad Sci U S A 98: 12890-12895. doi:10.1073/pnas.231320498. PubMed: 11606765. 11606765 PMC60795

[48] Kalinowski ST, Taper ML, Marshall TC (2007) Revising how the computer program CERVUS accommodates genotyping error increases success in paternity assignment. Mol Ecol 16: 1099-1106. doi:10.1111/j.1365-294X.2007.03089.x. PubMed: 17305863. 17305863

[49] Wroblewski EE, Murray CM, Keele BF, Schumacher-Stankey JC, Hahn BH et al. (2009) Male dominance rank and reproductive success in chimpanzees, *Pan* *troglodytes* *schweinfurthii* . Anim Behav 77: 873-885. doi:10.1016/j.anbehav.2008.12.014. PubMed: 19498952. 19498952 PMC2689943

[50] R Development Core Team (2012) R: A language and environment for statistical computing. Vienna, Austria: R Foundation for Statistical Computing.

[51] Newton-Fisher NE, Emery Thompson M, Reynolds V, Boesch C, Vigilant L (2010) Paternity and social rank in wild chimpanzees (*Pan* *troglodytes*) from the Budongo Forest, Uganda. Am J Phys Anthropol 142: 417–428. PubMed: 20033921. 20033921 10.1002/ajpa.21241

[52] Wrangham RW (1980) An ecological model of female-bonded primate groups. Behaviour: 262-300.

[53] Williams JM, Lonsdorf EV, Wilson ML, Schumacher-Stankey J, Goodall J et al. (2008) Causes of death in the Kasekela chimpanzees of Gombe National Park, Tanzania. Am J Primatol 70: 766-777. doi:10.1002/ajp.20573. PubMed: 18506732. 18506732

[54] Lukas D, Reynolds V, Boesch C, Vigilant L (2005) To what extent does living in a group mean living with kin? Mol Ecol 14: 2181-2196. doi:10.1111/j.1365-294X.2005.02560.x. PubMed: 15910336. 15910336

[55] De Lathouwers M, Van Elsacker L (2005) Reproductive parameters of female *Pan* *paniscus* and *P*. *Troglodytes*: Quality versus quantity. Int J Primatol 26: 55 - 71. doi:10.1007/s10764-005-0723-0.

[56] Cox DR (1972) Regression models and life-tables. J R Stat Soc B Stat Methodol 34: 187-220.

[57] Therneau T (2009) Package ‘coxme’. Mixed Effects Cox Models. Available: http://r-forge.r-project.org.

[58] Surbeck M, Hohmann G (2013) Intersexual dominance relationships and the influence of leverage on the outcome of conflicts in wild bonobos (Pan paniscus). Behav Ecol Sociobiol: 1-14.24436508

[59] Therneau T (2009) Package ‘survival’. Survival analysis, including penalised likelihood. Available: http://r-forge.r-project.org.

[60] Ellegren H (2000) Heterogeneous mutation processes in human microsatellite DNA sequences. Nat Genet 24: 400-402. doi:10.1038/74249. PubMed: 10742106. 10742106

[61] Silk JB, Alberts SC, Altmann J (2004) Patterns of coalition formation by adult female baboons in Amboseli, Kenya. Anim Behav 67: 573-582. doi:10.1016/j.anbehav.2003.07.001.

[62] Chapais B (2001) Primate nepotism: What is the explanatory value of kin selection? Int J Primatol 22: 203-229. doi:10.1023/A:1005619430744.

[63] Silk JB (2009) Nepotistic cooperation in non-human primate groups. Philos Trans R Soc Lond B Biol Sci 364: 3243-3254. doi:10.1098/rstb.2009.0118. PubMed: 19805431. 19805431 PMC2781876

[64] Strier KB, Chaves PB, Mendes SL, Fagundes V, Di Fiore A (2011) Low paternity skew and the influence of maternal kin in an egalitarian, patrilocal primate. Proc Natl Acad Sci U S A 108: 18915-18919. doi:10.1073/pnas.1116737108. PubMed: 22065786. 22065786 PMC3223441

[65] Surbeck M, Deschner T, Weltring A, Hohmann G (2012) Social correlates of variation in urinary cortisol in wild male bonobos (Pan paniscus). Horm Behav 62: 27-35. doi:10.1016/j.yhbeh.2012.04.013. PubMed: 22565126. 22565126

[66] Clay Z, de Waal FB (2013) Development of socio-emotional competence in bonobos. Proc Natl Acad Sci U S A 110: 18121-18126. doi:10.1073/pnas.1316449110. PubMed: 24127600. 24127600 PMC3831480

[67] Wobber V, Wrangham R, Hare B (2010) Bonobos exhibit delayed development of social behavior and cognition relative to chimpanzees. Curr Biol 20: 226-230. doi:10.1016/j.cub.2010.01.009. PubMed: 20116251. 20116251

[68] Wobber V, Wrangham R, Hare B (2010) Application of the heterochrony framework to the study of behavior and cognition. Commun Integr Biol 3: 337-339. doi:10.4161/cib.3.4.11762. PubMed: 20798819. 20798819 PMC2928311

[69] Tan J, Hare B (2013) Bonobos share with strangers. PLOS ONE 8: e51922. doi:10.1371/journal.pone.0051922. PubMed: 23300956. 23300956 PMC3534679

[70] Wobber V, Hare B, Maboto J, Lipson S, Wrangham R et al. (2010) Differential changes in steroid hormones before competition in bonobos and chimpanzees. Proc Natl Acad Sci U S A 107: 12457-12462. doi:10.1073/pnas.1007411107. PubMed: 20616027. 20616027 PMC2906573

[71] Hare B, Wobber V, Wrangham R (2012) The self-domestication hypothesis: evolution of bonobo psychology is due to selection against aggression. Anim Behav 83: 573-585. doi:10.1016/j.anbehav.2011.12.007.

[72] Kappeler PM, Pereira ME (2003) Primate Life Histories and Socioecology. Chicago: The University of Chicago Press.

[73] Bronikowski AM, Alberts SC, Altmann J, Packer C, Carey KD et al. (2002) The aging baboon: comparative demography in a non-human primate. Proc Natl Acad Sci U S A 99: 9591-9595. doi:10.1073/pnas.142675599. PubMed: 12082185. 12082185 PMC123185

[74] Gurven M, Kaplan H (2007) Longevity among hunter-gatherers: A cross-cultural examination. Popul Dev Rev 33: 321-365. doi:10.1111/j.1728-4457.2007.00171.x.

[75] Stumpf R (2007) Chimpanzees and bonobos: diversity within and between species. In: Campbell CJ Fuentes A MacKinnon KC Primates in Perspective. New York: Oxford University Press pp. 321-344.

[76] Williams GC (1957) Pleiotropy, natural selection, and the evolution of senescence. Evolution 11: 398-411. doi:10.2307/2406060.

[77] Medawar P (1952) An unsolved problem of biology. London: HK Lewis & Co.

[78] Furuichi T (2009) Factors underlying party size differences between chimpanzees and bonobos: a review and hypotheses for future study. Primates 50: 197-209. doi:10.1007/s10329-009-0141-6. PubMed: 19353234. 19353234

[79] Wittiger L, Boesch C (2013) Female gregariousness in Western Chimpanzees (Pan troglodytes verus) is influenced by resource aggregation and the number of females in estrus. Behav Ecol Sociobiol: 1-15.24436508

[80] Gibson MA, Mace R (2005) Helpful grandmothers in rural Ethiopia: A study of the effect of kin on child survival and growth. Evol Hum Behav 26: 469-482. doi:10.1016/j.evolhumbehav.2005.03.004.

[81] Sear R, Mace R, McGregor IA (2000) Maternal grandmothers improve nutritional status and survival of children in rural Gambia. Proc Biol Sci 267: 1641-1647. doi:10.1098/rspb.2000.1190. PubMed: 11467427. 11467427 PMC1690719

[82] Hawkes K (2003) Grandmothers and the evolution of human longevity. Am J Hum Biol 15: 380-400. doi:10.1002/ajhb.10156. PubMed: 12704714. 12704714

[83] Borgerhoff Mulder M (2007) Hamilton's rule and kin competition: the Kipsigis case. Evol Hum Behav 28: 299-312. doi:10.1016/j.evolhumbehav.2007.05.009.

[84] Fairbanks LA, McGuire MT (1986) Age, reproductive value, and dominance-related behaviour in vervet monkey females: cross-generational influences on social relationships and reproduction. Anim Behav 34: 1710-1721. doi:10.1016/S0003-3472(86)80258-5.

[85] Borries C (1988) Patterns of grandmaternal behaviour in free-ranging Hanuman langurs (*Presbytis* *entellus*). Hum Evol 3: 239-259. doi:10.1007/BF02435856.

[86] Clutton-Brock TH (1988) Reproductive Success; Clutton-Brock TH Chicago: University of Chicago Press.

[87] Widdig A (2007) Paternal kin discrimination: the evidence and likely mechanisms. Biol Rev Camb Philos Soc 82: 319-334. doi:10.1111/j.1469-185X.2007.00011.x. PubMed: 17437563. 17437563

[88] Lehmann J, Fickenscher G, Boesch C (2006) Kin biased investment in wild chimpanzees. Behaviour 143: 931-955. doi:10.1163/156853906778623635.

[89] Parr LA, de Waal FB (1999) Visual kin recognition in chimpanzees. Nature 399: 647-648. doi:10.1038/21345. PubMed: 10385114. 10385114

[90] Huchard E, Charpentier MJ, Marshall H, King AJ, Knapp LA et al. (2013) Paternal effects on access to resources in a promiscuous primate society. Behav Ecol 24: 229-236. doi:10.1093/beheco/ars158.

[91] Hohmann G (2009) The diets of non-human primates: frugivory, food processing, and food sharing. In: Hublin J-J Richards MP The Evolution of Hominin Diets: Integrating Approaches to the Study of Paleolithic Subsistence. London: Springer.

[92] Fruth B, Hohmann G (2002) How bonobos handle hunts and harvests: why share food? In: Boesch C Hohmann G Marchant L Behavioral Diversity in Chimpanzees and Bonobos. Cambridge: Cambridge University Press pp. 231-243.

