# Supplementary figures and images for: Co–Residence between Males and Their Mothers and Grandmothers Is More Frequent in Bonobos Than Chimpanzees
Source: PLoS One. 2013 Dec 17;8(12):e83870. doi: 10.1371/journal.pone.0083870 (PMC3866280; doi:10.1371/journal.pone.0083870)

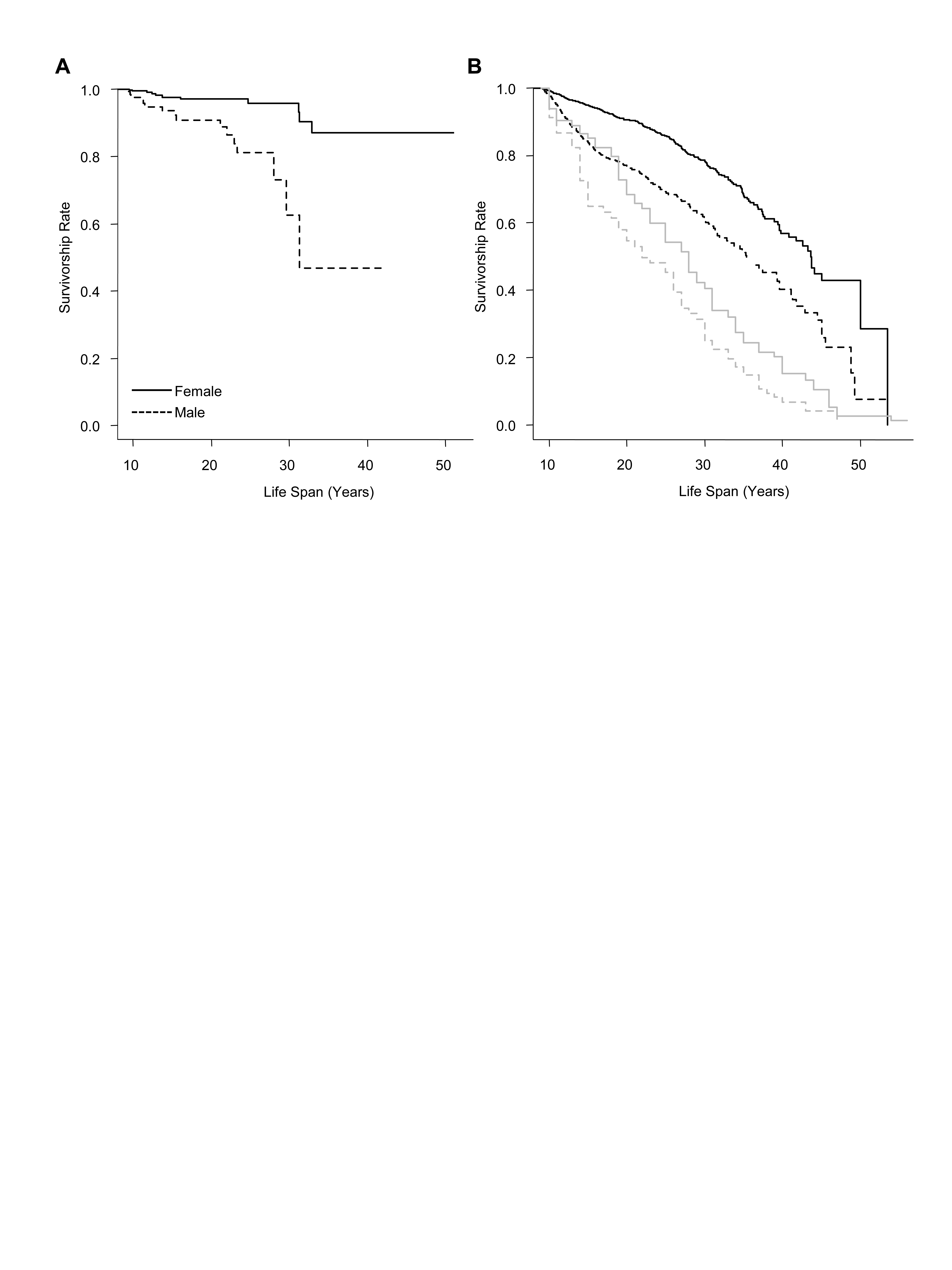

Supplement: Figure S1 — (A) Bonobo and (B) chimpanzee survivorship among captive females and males. In (B), for comparison, survivorship rates of wild chimpanzees published previously [32]are drawn in grey. In both species, captive females had higher rates of survival than captive males, however the difference was statistically significant only in chimpanzees (P – value from permutation procedure = 0.001), but not among bonobos (P – value from permutation procedure = 0.081). (TIF) [file pone.0083870.s001.tif]
