# Supplementary material for: Co–Residence between Males and Their Mothers and Grandmothers Is More Frequent in Bonobos Than Chimpanzees
Source: PLoS One. 2013 Dec 17;8(12):e83870. doi: 10.1371/journal.pone.0083870 (PMC3866280; doi:10.1371/journal.pone.0083870)
Supplement: Table S1 — Parentage assignments in the free–living LuiKotale Bompusa bonobo group and the Taï Middle and South Western chimpanzee groups. Parentage was determined from genotypes comprised of 19 autosomal loci. As our chimpanzee study spans several years, the age class of an individual might have changed over time. Maternal relationships known from behavior were confirmed in all infants and juveniles. All but one assignment met the 99% confidence criterion (see footnote). Females are written in capitals. n.a., not assigned. 1 Reported only for male offspring being adolescent or adult in study period 2; Reported for parent – pair comparisons with confirmed or assigned mother 3; One adolescent female and two juveniles were not genotyped 4; One mismatch (1 base pair indel common in that population) to assigned sire 5; One mismatch to mother at heterozygous locus (both alleles differ, one allele with one repeat unit difference to maternal allele), in triadic comparison with potential sires one mismatch to the assigned sire at heterozygous locus (one repeat unit difference to one paternal allele) 6; Paternity assigned at 95% confidence level 7; In triadic comparison one mismatch to the assigned sire at heterozygous locus (one repeat unit difference) 8; One mismatch to mother at heterozygous locus (both alleles differ, one allele with one repeat unit difference to maternal allele). (DOCX) [file pone.0083870.s002.docx]

**Table S1. Parentage assignments in the free–living LuiKotale Bompusa bonobo group and the Taï Middle and South Western chimpanzee groups.**

| **ID** | **Sex** | **Age class during study period** | **Mother** | **Sire** | **Mother – offspring mismatches** | **Mismatches to next best female^1^** | **Parent – pair – offspring mismatches** | **Mismatches to next best male^2^** |
| --- | --- | --- | --- | --- | --- | --- | --- | --- |
| **Bonobo^3^** |  |  |  |  |  |  |  |  |
| Egon | M | infant | EVA | Camillo | 0 |  | 0 | 3 |
| Hugo | M | infant | HANNAH | Tito | 0 |  | 0 | 3 |
| IDA | F | infant | IRIS | Tito | 0 |  | 0 | 2 |
| OLIVIA^4^ | F | infant | OLGA | Jack | 0 |  | 1 | 8 |
| PEGGY | F | Infant | PAULA | n.a. | 0 |  | n.a. | 3 |
| RITA | F | infant | RIO | Tito | 0 |  | 0 | 5 |
| Moe | M | juvenile | MARTHA | n.a. | 0 |  | n.a. | 5 |
| OPHELIA | F | juvenile | OLGA | n.a. | 0 |  | n.a. | 5 |
| Roque | M | juvenile | RIO | n.a. | 0 |  | n.a. | 6 |
| Apollo* | M | adolescent | HANNAH | n.a. | 0 | 5 | n.a. | 3 |
| Emil* | M | adolescent | EVA | n.a. | 0 | 4 | n.a. | 5 |
| Max* | M | adolescent | n.a. | n.a. | n.a. | 5 | n.a. | 2 |
| Pan* | M | adolescent | PAULA | n.a. | 0 | 5 | n.a. | 6 |
| Ben* | M | adult | ZOE | n.a. | 0 | 3 | n.a. | 6 |
| Camillo* | M | adult | MARTHA | n.a. | 0 | 3 | n.a. | 7 |
| Dante* | M | adult | n.a. | n.a. | n.a. | 3 | n.a. | 7 |
| Jack* | M | adult | n.a. | n.a. | n.a. | 4 | n.a. | 2 |
| Tito* | M | adult | MARTHA | n.a. | 0 | 4 | n.a. | 5 |
| **Chimpanzee – Taï Middle** | | | | | | | |  |
| JANINE | F | infant | JESSICA | Urs | 0 |  | 0 | 3 |
| Jonathan | M | infant | JESSICA | Bob | 0 |  | 0 | 5 |
| KASSIOPEÉ | F | infant | KADY | Léo | 0 |  | 0 | 4 |
| Noah | M | infant – juvenile | NADESH | Urs | 0 |  | 0 | 4 |
| KOULO | F | juvenile – adolescent | KADY | n.a. | 0 | 3 | n.a. | 4 |
| Bob | M | adult | n.a. | n.a. | n.a. | 3 | n.a. | 2 |
| Léo | M | adult | NADESH | n.a. | 0 | 2 | n.a. | 5 |
| UrsN | M | adult | n.a. | n.a. | n.a. | 3 | n.a. | 3 |
| **Chimpanzee – Taï South** | | | | | | | |  |
| AKROUBA | F | infant | ATRA | Sagu | 0 |  | 0 | 2 |
| ALINA | F | infant | ATRA | n.a. | 0 |  | n.a. | 2 |
| Caramel | M | infant | COCO | Sagu | 0 |  | 0 | 3 |
| Huxel | M | infant | HARAKA | Zyon | 0 |  | 0 | 3 |
| JAVA | F | infant | JULIA | Sagu | 0 |  | 0 | 2 |
| Kirikou | M | infant | KINSHASA | Woodstock | 0 |  | 0 | 6 |
| Lukas | M | infant | LOUISE | Kaos | 0 |  | 0 | 3 |
| Lula | M | infant | LOUISE | Sagu | 0 |  | 0 | 2 |
| OPHELIA^5^ | F | infant | OLIVIA | Zyon | 1 |  | 2 | 5 |
| Ravel^6^ | M | infant | RUBRA | Zyon | 0 |  | 0 | 2 |
| Téré^7^ | M | infant | TITA | Zyon | 0 |  | 1 | 3 |
| Voltaire | M | infant | VIRUNGA | Sagu | 0 |  | 0 | 5 |
| WALA | F | infant | WAPI | Besar | 0 |  | 0 | 3 |
| Wamba | M | infant | WAPI | Kaos | 0 |  | 0 | 4 |
| YAOUNDE | F | infant | YUCCA | Zyon | 0 |  | 0 | 4 |
| ZITA | F | infant | ZORA | Zyon | 0 |  | 0 | 3 |
| ZOE | F | infant | ZORA | Kaos | 0 |  | 0 | 5 |
| CÉLINE | F | infant – adolescent | COCO | Kaos | 0 |  | 0 | 3 |
| Kuba | M | infant – adolescent | KABISHA | Zyon | 0 |  | 0 | 4 |
| ATHÉNA | F | infant – juvenile | ATRA | Zyon | 0 |  | 0 | 3 |
| ENDORA | F | infant – juvenile | EVA | Mkubwa | 0 |  | 0 | 5 |
| Ibrahim | M | infant – juvenile | ISHA | Zyon | 0 |  | 0 | 3 |
| Inousha | M | infant – juvenile | ISHA | n.a. | 0 |  | n.a. | 5 |
| Jacobo | M | infant – juvenile | JULIA | n.a. | 0 |  | n.a. | 5 |
| Mustapha | M | infant – juvenile | MARGOT | n.a. | 0 |  | n.a. | 4 |
| Oreste | M | infant – juvenile | OLIVIA | n.a. | 0 |  | n.a. | 3 |
| RÉBECCA | F | infant – juvenile | RUBRA | n.a. | 0 |  | n.a. | 2 |
| Romario | M | infant – juvenile | RUBRA | Kaos | 0 |  | 0 | 3 |
| SETTUT | F | infant – juvenile | SUMATRA | Kaos | 0 |  | 0 | 4 |
| Shogun^8^ | M | infant – juvenile | SUMATRA | Zyon | 1 |  | 1 | 6 |
| Yao | M | infant – juvenile | YUCCA | Zyon | 0 |  | 0 | 4 |
| Max | M | juvenile | MANDY | Kaos | 0 |  | 0 | 5 |
| Olduvai | M | juvenile | OLIVIA | n.a. | 0 |  | n.a. | 4 |
| Linus | M | juvenile – adolescent | LOUISE | n.a. | 0 | 2 | n.a. | 5 |
| Utan | M | juvenile – adolescent | n.a. | n.a. | n.a. | 3 | n.a. | 3 |
| Woodstock | M | juvenile – adolescent | WAPI | n.a. | 0 | 4 | n.a. | 5 |
| Gogol | M | juvenile– adult | GARUDA | n.a. | 0 | 7 | n.a. | 2 |
| Taboo | M | juvenile– adult | TITA | n.a. | 0 | 5 | n.a. | 4 |
| Yoghiti | M | adolescent | YUCCA | n.a. | 0 | 5 | n.a. | 4 |
| Besar | M | adolescent – adult | n.a. | n.a. | n.a. | 2 | n.a. | 2 |
| Sagu | M | adolescent – adult | SUMATRA | n.a. | 0 | 4 | n.a. | 4 |
| Kaos | M | adult | n.a. | n.a. | n.a. | 6 | n.a. | 2 |
| Zyon | M | adult | n.a. | n.a. | n.a. | 5 | n.a. | 4 |

Parentage was determined from genotypes comprised of 19 autosomal loci. As our chimpanzee study spans several years, the age class of an individual might have changed over time. Maternal relationships known from behavior were confirmed in all infants and juveniles. All but one assignment met the 99% confidence criterion (see footnote). Females are written in capitals. n.a., not assigned.

^1^ Reported only for male offspring being adolescent or adult in study period; ^2^ Reported for parent – pair comparisons with confirmed or assigned mother; ^3^ One adolescent female and two juveniles were not genotyped; ^4^ One mismatch (1 base pair indel common in that population) to assigned sire; ^5^ One mismatch to mother at heterozygous locus (both alleles differ, one allele with one repeat unit difference to maternal allele), in triadic comparison with potential sires one mismatch to the assigned sire at heterozygous locus (one repeat unit difference to one paternal allele); ^6^ Paternity assigned at 95% confidence level; ^7^ In triadic comparison one mismatch to the assigned sire at heterozygous locus (one repeat unit difference); ^8^ One mismatch to mother at heterozygous locus (both alleles differ, one allele with one repeat unit difference to maternal allele).
