# Supplementary material for: Co–Residence between Males and Their Mothers and Grandmothers Is More Frequent in Bonobos Than Chimpanzees
Source: PLoS One. 2013 Dec 17;8(12):e83870. doi: 10.1371/journal.pone.0083870 (PMC3866280; doi:10.1371/journal.pone.0083870)
Supplement: Table S2 — Results from a Cox mixed–effects model examining survival in captive female and male bonobos and chimpanzees. Sex (female; male), Location status (permanently housed in zoological facilities; non-permanently housed in zoological facilities), Transfer (number and timing of individuals' transfers between locations) and Entry age (age of individuals when entering each time interval between subsequent transfers) were included as fixed effects (the two latter time–dependent). A P – value for Sex was derived from a permutation procedure. *Estimate of the regression coefficient. **Samples sizes: female = 329, female deaths = 11; male = 129, male deaths=16. ***Samples sizes: female = 2427, female deaths = 287; male = 811, male deaths = 220. (DOCX) [file pone.0083870.s003.docx]

**Table S2. Results from a Cox mixed–effects model examining survival in captive female and male bonobos and chimpanzees.**

|  | Estimate* | Std. Error | *z* | *P* – value |
| --- | --- | --- | --- | --- |
| **Bonobo survival** |  |  |  |  |
| Location status | 0.00 | 0.53 | 0 |  |
| Sex** | 0.66 | 0.51 | 1.28 | 0.081 |
| Transfer | 0.20 | 0.20 | 1 |  |
| Entry age | –0.15 | 0.05 | –3.28 |  |
| **Chimpanzee survival** |  |  |  |  |
| Location status | 0.00 | 0.24 | –0.01 |  |
| Sex*** | 0.64 | 0.23 | 2.72 | 0.001 |
| Transfer | 0.10 | 0.09 | 1.16 |  |
| Entry age | –0.05 | 0.01 | –4.06 |  |

*Sex* (female; male), *Location status* (permanently housed in zoological facilities; non–permanently housed in zoological facilities), *Transfer* (number and timing of individuals' transfers between locations) and *Entry age* (age of individuals when entering each time interval between subsequent transfers) were included as fixed effects (the two latter time–dependent). A *P* – value for *Sex* was derived from a permutation procedure.

*Estimate of the regression coefficient. **Samples sizes: female = 329, female deaths = 11; male = 129, male deaths=16. ***Samples sizes: female = 2427, female deaths = 287; male = 811, male deaths = 220.
